# Genetic diversity, transmission dynamics and drug resistance of M*ycobacterium tuberculosis* in Angola

**DOI:** 10.1038/srep42814

**Published:** 2017-02-23

**Authors:** João Perdigão, Sofia Clemente, Jorge Ramos, Pedro Masakidi, Diana Machado, Carla Silva, Isabel Couto, Miguel Viveiros, Nuno Taveira, Isabel Portugal

**Affiliations:** 1 iMed.ULisboa – Instituto de Investigação do Medicamento, Faculdade de Farmácia, Universidade de Lisboa, Lisboa, Portugal; 2 Hospital da Divina Providência, Serviço de Doenças Infecciosas, Luanda, Angola; 3 Unidade de Microbiologia Médica, Global Health and Tropical Medicine, GHTM, Instituto de Higiene e Medicina Tropical, IHMT, Universidade Nova de Lisboa, UNL, Lisboa, Portugal; 4 Centro de Investigação Interdisciplinar Egas Moniz, Instituto Superior de Ciências da Saúde Egas Moniz, Monte de Caparica, Portugal

## Abstract

Tuberculosis (TB) poses a serious public health problem in Angola. No surveillance data on drug resistance is available and nothing is known regarding the genetic diversity and population structure of circulating *Mycobacterium tuberculosis* strains. Here, we have genotyped and evaluated drug susceptibility of 89 *Mycobacterium tuberculosis* clinical isolates from Luanda. Thirty-three different spoligotype profiles corresponding to 24 different Shared International Types (SIT) and 9 orphan profiles were detected. SIT 20 (LAM1) was the most prevalent (n = 16, 18.2%) followed by SIT 42 (LAM9; n = 15, 17.1%). Overall, the *M. tuberculosis* population structure in this sample was dominated by LAM (64.8%) and T (33.0%) strains. Twenty-four-*loci* MIRU-VNTR analysis revealed that a total of 13 isolates were grouped in 5 distinct clusters. Drug susceptibility data showed that 22 (24.7%) of the 89 clinical isolates were resistant to one or more antibacillary drugs of which 4 (4.5%) were multidrug resistant. In conclusion, this study demonstrates a high predominance of LAM strains circulating in the Luanda setting and the presence of recent transmission events. The rate and the emergence dynamics of drug resistant TB found in this sample are significant and highlight the need of further studies specifically focused on MDR-TB transmission.

Despite the importance that the African region plays in a global TB epidemiological context, many countries still lack data on the prevalence of specific *M. tuberculosis* strains and drug resistance^1^. This is the case for Angola, which presently lacks any data concerning drug resistance rates and prevalence of specific *Mycobacterium tuberculosis* genotypes and respective population structure. The latest World Health Organization (WHO) estimates for Angola suggest the occurrence of 90 000 new cases and an incidence rate of 370 cases per 100 000 habitants in 2014 combined with an increasing trend over the last two decades^1^. Furthermore, these estimates point towards the occurrence of 1500 cases of multidrug resistant (MDR) TB that were not yet bacteriologically confirmed^1^.

The situation is particularly concerning in Luanda, the capital city of Angola, and its province, which accounts for approximately one-third of the country’s TB cases (21 281 cases notified in 2013)^2^,^3^.

Understanding how TB transmission takes place is a key component to strategically manage TB from a public health perspective^4^,^5^,^6^. Furthermore, given the unexpected degree of genetic diversity more recently unveiled by whole genome sequencing and since different strains have the ability to elicit distinct immunopathological events, tracking specific strains plays an even more important role^7^,^8^,^9^,^10^. Several typing methods have been developed over the past decades to investigate the clonality, population structure and transmission of *M. tuberculosis*^5^,^11^,^12^,^13^,^14^. These, have gradually led to the development of online databases and have enabled the identification of epidemiologically links between patients and risk factors that otherwise would be nearly impossible to identify with the use of traditional epidemiological investigation and contact tracing^6^,^15^,^16^.

Currently, there is no data concerning *M. tuberculosis* diversity, population structure and drug resistance in Angola. Herein, we have characterized the genetic diversity and drug susceptibility profiles of circulating *M. tuberculosis* strains recovered from patients followed at a central hospital in Luanda.

## Methods

### Patients and Clinical Isolates

A total of 106 sputum samples, positive for sputum smear acid-fast bacilli, were collected from patients clinically diagnosed with TB in Hospital da Divina Providência (HDP) in Luanda district. HDP is located in the Kilamba-Kiaxi municipality and serves an estimated population of 990 892 inhabitants. Regarding TB treatment, HDP has a unit dedicated to the diagnosis of respiratory diseases (approximately 600 new TB cases/year) and directly observed treatment. Sample collection was performed between March to June 2014, where all sputum smear positive samples, comprising approximately 18% of the hospitals yearly diagnosed cases, were collected to avoid unbiased selection criteria. Patients’ demographical and clinical data were collected from clinical records. The study was approved by the Angolan Ministry of Health’s Ethics Committee, and all methods were performed in accordance with the relevant guidelines and regulations, including informed consent from all patients enrolled in the study.

Sputum sample decontamination was carried out using the NaOH/N-acetyl-L-Cysteine method and cultured on Lowenstein-Jensen slants and Middlebrook 7H9 medium supplemented with oleic acid, albumin, dextrose and catalase (OADC) and an antibiotic cocktail comprised by carbenicillin (final concentration: 5 μg/ml), trimethoprim (15 μg/ml), amphotericin B (1 μg/ml) and polymyxin B (20 U/ml).

High molecular weight genomic DNA was extracted from mycobacterial grown on Lowenstein-Jensen media by the cetyltrimethylammonium bromide (CTAB) method^17^.

All isolates were identified as belonging to the *M. tuberculosis* complex by positive PCR amplification of an internal IS*6110* fragment^17^.

### Drug Susceptibility Testing (DST)

All *M. tuberculosis* complex isolates were tested for first-line DST to all first-line antibacillary drugs (isoniazid (INH), rifampicin (RIF), ethambutol (EMB), streptomycin (STP) and pyrazinamide (PZA)) through the BACTEC™ MGIT™ 960 system (Becton Dickinson Diagnostic Systems, Sparks, MD, USA) using the standardized procedure according to the manufacturer’s instructions^18^.

### Spoligotyping and MIRU-VNTR typing

Spoligotyping was performed as described previously by Kamerbeek *et al*.^12^. Detection of the hybridization patterns was carried out using the ECL^®^ Chemiluminescence Detection System (GE Healthcare^®^, Cleveland, OH, USA).

All isolates were typed by 24-*loci* MIRU-VNTR using the multiplex amplification procedure described by Supply *et al*.^11^.

Spoligotyping and MIRU-VNTR profiles were assigned to lineage, clade, shared international type (SIT) and MIRU International Type (MIT) using the SITVIT WEB international database (http://www.pasteur-guadeloupe.fr:8081/SITVIT_ONLINE/index.jsp) and/or the SPOTCLUST tool (http://tbinsight.cs.rpi.edu/run_spotclust.html)^16^,^19^.

A dendrogram was constructed based on the MIRU-VNTR and spoligotyping data, as appropriated, using the MIRU-VNTR*plus* international database^15^. A cluster was defined as group of two or more strains with identical profile. The Hunter-Gaston index of diversity was computed as described previously^20^.

A minimum spanning tree was also constructed using the MIRU-VNTR*plus* database to investigate phylogenetic relationships within the sample and identify clonal complexes. A clonal complex was defined as groups of isolates that are within dual-*locus* variants of each other.

All statistical analyses were conducted using the IBM^©^ SPSS^©^ Statistics v.21 (IBM Corporation, Armonk, NY, USA).

## Results

### Patients and Clinical Isolates

**Figure 1:**
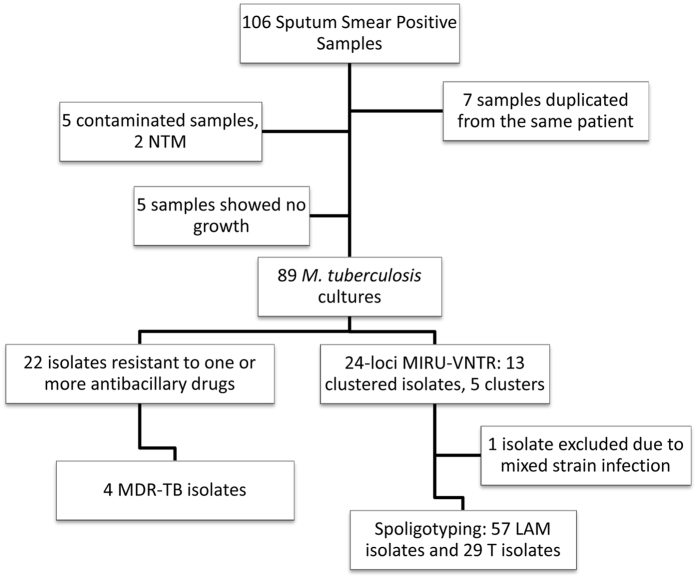
Flowchart illustrating the selection of the studied sample of *M. tuberculosis* clinical isolates and, main results from drug susceptibility testing and genotyping. From the 106 sputum samples positive for acid-fast bacilli, 7 were excluded since they were duplicates from the same patient, 5 were contaminated and another 5 showed no growth, leaving a total of 89 *M. tuberculosis* clinical isolates. Drug susceptibility testing revealed that 22 isolates were resistant to one or more antibacillary drugs, of which four corresponded to MDR-TB isolates. Genotyping analysis allowed the identification of 13 clustered isolates across five different MIRU-VNTR clusters with one isolate excluded due to mixed strain infection. Spoligotyping analysis revealed a population structure dominated mostly by LAM and T strains.

In the present study we have collected 106 sputum samples, positive for acid-fast bacilli, in HDP in 2014. After culture and identification, five samples were excluded due to contamination; seven samples were duplicated from the same patient; and, five samples showed no growth after a 3 month incubation period. The remaining samples yielded 89 *M. tuberculosis* complex clinical isolates, each corresponding to a different patient (Fig. 1).

It was only possible to collect partial demographical and clinical data for 60 (67.4%) of the 89 studied patients (see Supplementary Table 1). The majority of the patients resided in the Kilamba-Kiaxi municipality (n = 34/58, 58.6%), where HDP is located, followed by a significant proportion of patients living in Viana (n = 19/58, 32.8%) (Supplementary Table 1).

The majority of the patients comprising this sample were registered as new patients (n = 53/60, 88.3%), approximately half presented with cavitary lung disease (n = 29/50, 58.0%) and eight out of 55 patients (14.5%) were co-infected with HIV. Fifty-one (87.9%) out of 58 patients started the 2HRZE/4HR treatment regimen while the remaining seven started the 2HRZES/4HR treatment regimen. The majority of the patients (n = 39/60, 65.0%) achieved both bacteriological and clinical cure after completing the treatment regimen (Supplementary Table 1).

### Drug Resistance

We have detected eight different drug-resistance profiles and overall, 22 (24.7%) isolates showed resistance to one or more antibacillary drugs (Table 1). Four (4.5%) isolates exhibited a multidrug resistant (MDR) profile, i.e. resistance to at least isoniazid (INH) and rifampicin (RIF), whereas 13 of the 22 drug resistant isolates were resistant to INH or RIF and can thus be considered pre-MDR-TB isolates.

### Population structure and Molecular Epidemiology

**Figure 2:**
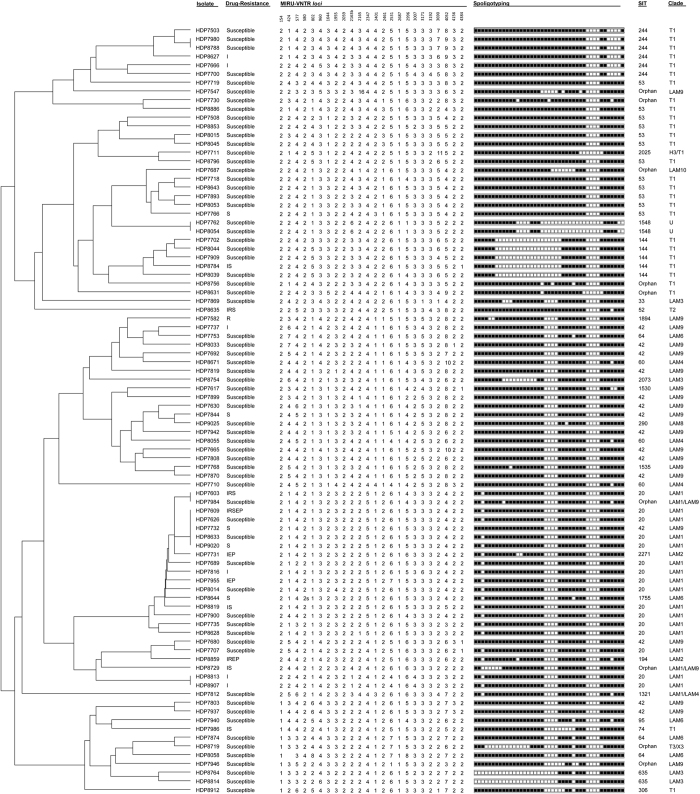
MIRU-VNTR dendrogram (24 *loci*) of the 88 *M. tuberculosis* clinical isolates analysed in the present study. Drug resistance: I – isoniazid; R – rifampicin; S – streptomycin; E – ethambutol; and, P – pyrazinamide.

To gain insight on the sample’s population structure all strains were genotyped by spoligotyping and 24-*loci* MIRU-VNTR. One isolate (HDP7743) showed double alleles in 5 of the 24 MIRU-VNTR *loci* and was therefore excluded from the genotypic analysis. Spoligotyping analysis of the remaining 88 *M. tuberculosis* isolates enabled the detection of 33 different spoligotype profiles corresponding to 24 different Shared International Types (SIT) and 9 orphan profiles (Table 2, Fig. 2). SIT 20 (LAM1) was the most prevalent SIT found (n = 16, 18.2%) followed by SITs 42 (LAM9; n = 15, 17. 1%) and 53 (T1; n = 12, 13.6%).

Comparatively, the 24-*loci* MIRU-VNTR analysis showed, as expected, a superior discriminatory power in which only 13 isolates were clustered across 5 clusters (Fig. 2, Table 2). Analysis of the genotypic data using the 12, 15 and 24-*loci* MIRU-VNTR sets showed that the 15- and 24-*loci* sets had comparable discriminatory ability as illustrated by the comparable values of the Hunter-Gaston Index of diversity (*D*) (Table 3)^20^,^21^. Both spoligotyping and 12-*loci* MIRU-VNTR, owing to a lesser capability of genotypic discrimination, yielded a high degree of clustering, which does not necessarily illustrate recent transmission events but rather be associated with an earlier common origin followed by diversification at the genotypic level, in particular at *loci* with a faster molecular clock^11^.

Drug resistant isolates were found across distinct clades and MIRU-VNTR clusters although the largest MIRU-VNTR cluster detected (n = 5) harboured 3 drug resistant isolates, including one MDR isolate (Fig. 1). A two-isolate cluster composed by pre-MDR strains (mono-INH resistance) was also detected.

**Figure 3:**
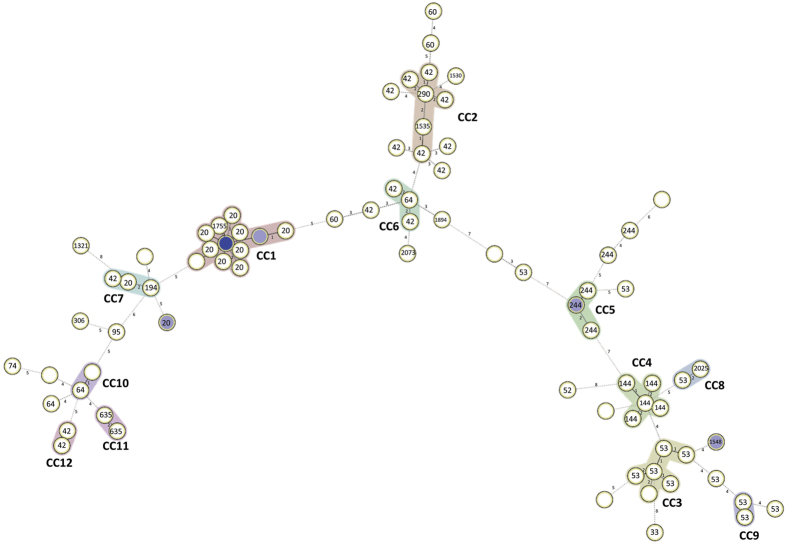
Minimum spanning tree of the 88 *M. tuberculosis* clinical isolates studied herein. The tree was constructed based on 24-*loci* MIRU-VNTR genotypic data and clonal complexes defined as MIRU-VNTR profiles within double-*locus* variants of each other. Clonal complexes have been highlighted and annotated on the tree (CC1-12) along with each node SIT, whenever each node is composed by strains of the same SIT and the spoligotype profile is not orphan. Numbers on the lines connecting each node indicate the *loci* difference between each node.

To better understand transmission, a minimum spanning tree was constructed using double-*locus* variants from 24-*loci* MIRU-VNTR data as a maximum distance to link and define clonal complexes (Fig. 3). We have identified 12 clonal complexes which ranged in size from two to 17 isolates. The largest clonal complex, CC1, was comprised by 17 LAM isolates, mostly belonging to the LAM1 clade and MIT10 (Supplementary Table 2). A second large LAM9 clonal complex, CC2, was also detected comprised by six isolates. T strains were mostly detected on three clonal complexes, CC3–6, comprised by 6, 5 and 4 isolates, respectively. Furthermore, 32 singleton profiles were identified (Table 4).

Next, we have combined the genotypic data with the geographical data, *i.e.* patient residency area. Geographical case dispersion analysis was restricted to the Kilamba-Kiaxi and Viana since all but 5 patients resided in these municipalities. This is expected since HDP is located in the eastern zone of Kilamba-Kiaxi, near the Viana municipality and primarily serves these two municipalities. Although not meeting statistical significance, genotypic dispersion through these municipalities appears to exhibit substantial differences for some of the clades or clonal complexes assessed by spoligotyping or MIRU-VNTR, respectively (Table 5). Generally, LAM strains were more prevalent in Kilamba-Kiaxi in comparison to the Viana municipality (LAM: 70.59% vs 42.11%, respectively), which consequently holds a higher percentage of T strains (52.63%). Stratification by clonal complex, clade and SIT allowed picturing the distribution of these genetic groups at a finer resolution: in Kilamba-Kiaxi CC1 stands out as the most prevalent MIRU-VNTR clonal complex, which correlates with a high SIT20/LAM1 prevalence in this municipality (Table 5). CC3 also appears to play an important role in Kilamba-Kiaxi’s epidemiological context, consistent with the prevalence of T1 strains. Although no clonal complex appears to be more prevalent in Viana, SIT53/T1 and SIT42/LAM9 strains exhibited a higher prevalence in this setting than in Kilamba-Kiaxi (Table 5).

## Discussion

In the present study we made the first characterization of the genetic diversity and drug resistance of *M. tuberculosis* complex strains circulating in Luanda, Angola’s most important setting concerning TB epidemiology^2^.

Drug susceptibility testing revealed a worrying situation concerning resistance rates. Approximately one-quarter of the studied isolates were resistant to one or more antibacillary drugs. This situation is particularly notorious for INH resistance which was detected on 18% of the clinical isolates. Pre-MDR-TB strains (*i.e.* monoresistant to INH or RIF) were found to represent 14.6% of the studied samples highlighting an increased potential for MDR-TB development during the course of treatment by resistance amplification due to standardized treatment regimens and particularly, in cases of poor adherence^22^,^23^. The potential risk for resistance amplification is well patent in the Lisboa3 and Q1 strains in Portugal, or the KZN strains in South Africa that have gradually acquired resistance to an increasing number of drugs over the years in a stepwise manner^8^,^24^,^25^. Also, the MDR-TB rate appears to be moderate (4.5%) but may in fact represent a serious problem from a public health perspective. Assuming that the rate found in this study is the same across the entire province setting, new MDR-TB cases can be estimated to be at least around 958 cases based on the official province data estimate for 2013, of 21 281 new cases/year^2^. This situation clearly contrasts with the data reported in a study conducted in the Ndola district, in Zambia, a neighbouring country, in which from 193 clinical isolates only 8.8% and 0.5% showed resistance to any antibacillary drug or MDR, respectively^26^. A clear example of the consequences of not having an adequate laboratory support on the follow up of TB patients under treatment, a recurrent situation in many countries with unreported high levels of acquired MDR-TB^27^,^28^,^29^.

Besides drug resistance we also investigated the *M. tuberculosis* genetic diversity, population structure and transmission dynamics. Genotypic analysis through spoligotyping and 24-*loci* MIRU-VNTR revealed a population structure dominated by LAM and the ill-defined T strains. Comparing our data with the recently published global framework for the LAM lineage proposed by Mokrousov *et al*.^30^ and the SNP barcode system for phylogenetic classification proposed by Coll *et al*.^10^, we find that: (i) the single strain belonging to SIT33 exhibits the VNTR signature characterized by *loci* 2401_2 and 3171_1 alleles in agreement to what has been proposed previously; (ii) 11 (12.5%) isolates bearing LAM or possibly LAM-derived spoligotype profiles formed a monophyletic clade bearing the *locus* 154_1 allele, characteristic of the RD115-sublineage/sublineage 4.3.3; 44 (50.0%) isolates bearing LAM profiles were found to exhibit the VNTR locus 802_1 allele proposed to be a marker for the RD174-sublineage/sublineage 4.3.4^10^,^30^. The data herein, presented therefore contributes to the VNTR data available for sub-Saharan Africa and is consistent with an increasing LAM prevalence when moving southwards on the African continent^30^.

It is also important to consider when structuring *M. tuberculosis* samples by spoligotyping clades that spoligotyping profiles show a tendency to converge and that a given clade does not necessarily share a more recent common ancestor. Such is particularly true, *e.g.*, among the ill-defined T lineage and LAM9 clade^10^. From a public health standpoint such polyphyletic clades may therefore enclose a higher degree of genetic diversity and underpin distinct transmission networks.

Again, when comparing with the neighbouring countries, only Zambia and Namibia have sufficient data on SITVIT WEB database to allow such comparison, as the *M. tuberculosis* population structure in the Democratic Republic of the Congo is presently unknown^16^. While Zambia exhibits a more diverse population structure dominated by the LAM11-ZWE (not found in the present study) but including other LAM, CAS, MANU and EAI strains, Namibia exhibits a more restricted diversity mainly dominated by SIT 20 (LAM1) strains that account to 78.5% of the strains deposited in SITVIT WEB for this country^16^. Although we cannot rule out epidemiological influence from the Democratic Republic of the Congo in the Angolan population structure, for Zambia there is no data supporting any epidemiological influence in Angola since no LAM11-ZWE strains were found in our sample. On the other hand, Namibia’s *M. tuberculosis* population structure might be or have been of importance in shaping the current Angolan epidemiological context concerning genetic diversity as the most prevalent SIT in Namibia (SIT 20) was also the most prevalent SIT in our study albeit with a significant difference in prevalence (78.5% vs 18.2%, respectively). The relations that both countries have maintained during the Angolan civil war might have been of particular importance in strain circulation between both countries as a peak of 30 881 Angolan citizens took refuge in Namibia in 2001, most of which have later returned to the country of origin^31^. Similarly, the Democratic Republic of the Congo, for which no data on the *M. tuberculosis* genetic diversity presently exists, hosted up to 186 879 Angolan refugees in the same period, which is likely to have influenced the *M. tuberculosis* population structure on both countries. Strain spreading from the Democratic Republic of the Congo, and eventually from Republic of Congo, is plausible given the documented spread of HIV-1 variants from these countries to Angola^32^.

However, another factor shaping the population structure found herein might be located further away, pertaining the Community of Portuguese Speaking Countries. In fact a very similar *M. tuberculosis* population structure is found in Portugal when it comes to the prevalence of LAM strains^16^. All SIT20/LAM1 isolates (n = 16, 18.2%), and derived SITs, bear the VNTR *locus* 2461_2 allele while Lisboa3 clade strains in Portugal harbour the 2461_1 allele, which on the one hand hampers the establishment of a more direct link but, on the other hand, makes it possible to hypothesize that Lisboa3 strains might have derived from such SIT20/LAM1 strains bearing the 2461_2 allele during its evolutionary trajectory towards XDR-TB^8^,^30^. Such hypothesis warrants, however, further studies as the SIT20/LAM1 strains found in this study might have originated in Latin America as well^16^,^30^. Furthermore, 13 (18.8%) isolates were also found to harbour the VNTR *locus* 1644_1*_*allele that according to Mokrousov *et al*.^30^ is only observed in few isolates across the RD174-sublineage with the exception of Q1 (SIT1106/LAM4) clade strains. This latter finding also raises the hypothesis of a common evolutionary origin between these strains, and countries, that merits further investigation^8^,^30^. Historical ties connect the Community of the Portuguese Speaking Countries, which likely favour, at a macroepidemiological level, an enhanced strain circulation between these countries.

There is some paucity of data concerning clustering rates in African countries. The clustering rates obtained in this study, particularly with the 15 or 24-*loci* MIRU-VNTR sets, are comparable with the ones reported by Homolka *et al*.^33^ in a study involving strains from Sierra Leone but considerably lower than the clustering rates obtained by Mulenga *et al*.^34^ in a study from Ndola district in Zambia^33^,^34^. It is also likely that the clustering rate in our study might be underestimated due the restricted sampling timeframe^34^. Overall, the population structure provided by MIRU-VNTR typing was consistent with the spoligotyping population structure. Nevertheless, as reported previously, MIRU-VNTR allows a deeper discrimination between isolates and is therefore more adequate for epidemiological surveillance^11^. Still in this regard, no significant difference exists between the discriminatory ability of 24 *vs* the 15 MIRU-VNTR *loci* sets as measured by the Hunter and Gaston Index of Diversity (0.996 vs 0.993, respectively) for this setting. For routine epidemiological surveillance the 15-*loci* set is therefore recommended with the 24-*loci* set being more adequate for *M. tuberculosis* phylogenetic studies, as proposed by Supply *et al*.^11^.

Besides demonstrating the presence of recent transmission events, complex transmission chains with a common origin may be underlying local strain diversification. In particular, the genotypic diversification exhibited by CC1, in comparison with the other clonal complexes, and its association with SIT20/LAM1 clades suggest an earlier introduction of LAM1 strains in this setting when compared with other clade-associated clonal complexes of restricted genotypic diversification. Concerning its global importance, SIT20/LAM1/MIT10 strains have already been previously described in Portugal as associated with drug resistance, highlighting, again, the putative epidemiological links between Portuguese-speaking countries^8^.

Additionally, given the high number of singleton profiles and non-clustered isolates, another important aspect to consider is that TB reactivation may be playing an important role in this setting’s TB epidemiology. Tackling TB by preventive treatment on specific sub-populations associated with known risk factors for disease reactivation may contribute to slowing down the country’s increasing TB incidence^35^.

In conclusion, this first cross-sectional study conducted in Luanda, Angola, provides a framework for future studies and programmatic management of TB in Angola. We provide sufficient evidence for cluster-based transmission with differential geographic dispersion. Furthermore, the moderate rate of MDR-TB found in this sample has major public health implications and highlights the need for further studies specifically focused on MDR-TB transmission.

## Additional Information

**How to cite this article**: Perdigão, J. *et al*. Genetic diversity, transmission dynamics and drug resistance of M*ycobacterium tuberculosis* in Angola. *Sci. Rep.* **7**, 42814; doi: 10.1038/srep42814 (2017).

**Publisher's note:** Springer Nature remains neutral with regard to jurisdictional claims in published maps and institutional affiliations.

## Supplementary Material

Supplementary Tables

## Figures and Tables

**Table 1:** First-line drug resistance profiles found among the 89 studied isolates.

Resistance Profile^1^	No. of Isolates (%)
I	6 (6.74)
IEP	2 (2.25)
IREP	1 (1.12)
IRS	2 (2.25)
IRSEP	1 (1.12)
IS	4 (4.49)
R	1 (1.12)
S	5 (5.62)
Susceptible	67 (75.28)
Total	89 (100.00)

^1^Resistance profile: I, isoniazid; R, rifampicin; S, streptomycin; E, ethambutol; P, pyrazinamide.

**Table 2:** Number of isolates found in the present study by SIT and clade.

Spoligotyping			No. Isolates (%)
SIT	Clade	Octal Code	
635	LAM3	000000007560771	2 (2.27)
Orphan	LAM1/LAM9	601777607760771	1 (1.14)
Orphan	LAM1/LAM9	676777607760731	1 (1.14)
2271	LAM2	677717607760771	1 (1.14)
194	LAM2	677737607760731	1 (1.14)
1755	LAM6	677777607560771	1 (1.14)
1321	LAM1/LAM4	677777607760731	1 (1.14)
20	LAM1	677777607760771	16 (18.18)
Orphan	T3/X3	700003607560371	1 (1.14)
1894	LAM9	747777607760771	1 (1.14)
144	T1	770000003760771	5 (5.68)
2073	LAM3	776000607760771	1 (1.14)
33	LAM3	776177607760771	1 (1.14)
1535	LAM9	777577607760771	1 (1.14)
1548	LAM8	777703400000360	2 (2.27)
Orphan	T1	777737737760731	1 (1.14)
Orphan	LAM9	777777405720771	1 (1.14)
Orphan	T1	777777557560771	1 (1.14)
Orphan	T1	777777577740371	1 (1.14)
74	T1	777777600160771	1 (1.14)
Orphan	LAM9	777777601460771	1 (1.14)
306	T1	777777601560771	1 (1.14)
290	LAM8	777777606760771	1 (1.14)
95	LAM6	777777607560731	1 (1.14)
64	LAM6	777777607560771	3 (3.41)
1530	LAM4	777777607760711	1 (1.14)
60	LAM4	777777607760731	3 (3.41)
42	LAM9	777777607760771	15 (17.05)
Orphan	LAM10	777777740160771	1 (1.14)
2025	Haarlem3/T1	777777777700371	1 (1.14)
244	T1	777777777760601	6 (6.82)
52	T2	777777777760731	1 (1.14)
53	T1	777777777760771	12 (13.64)
		Total	88 (100)

**Table 3:** Comparison of various diversity estimators across different MIRU-VNTR *loci* sets and spoligotyping.

Estimator^1^	MIRU-VNTR Set			Spoligotyping
	12-*loci*	15-*loci*	24-*loci*	
*D*	0.973	0.993	0.996	0.915
No. of clusters	17	5	5	9
No. of clustered isolates	53	15	13	64

^1^*D* – Hunter-Gaston Index of diversity.

**Table 4:** Clinical isolates present in each clonal complex defined as groups of 24-*loci* MIRU-VNTR profiles within double-*locus* variants of each other.

Clonal Complex	Isolates	No. of Isolates (%)	Clade (No. of Isolates)
CC1	HDP7609, HDP7626, HDP7732, HDP8633, HDP9020, HDP7609, HDP8819, HDP7689, HDP7816, HDP7955, HDP8014, HDP8644, HDP7735, HDP8628, HDP7603, HDP7984, HDP7900, HDP7731	17 (19.3)	LAM1 (16), LAM6 (1)
CC2	HDP9025, HDP7630, HDP7942, HDP7844, HDP7768, HDP7870	6 (6.8)	LAM9 (5), LAM8 (1)
CC3	HDP8643, HDP7766, HDP7687, HDP7718, HDP8053, HDP7893	6 (6.8)	T1 (5), LAM10 (1)
CC4	HDP8044, HDP7909, HDP8039, HDP8784, HDP7702	5 (5.6)	T1 (5)
CC5	HDP7980, HDP8788, HDP7503, HDP8627	4 (4.5)	T1 (4)
CC6	HDP7753, HDP8033, HDP7737	3 (3.4)	LAM9 (2), LAM6 (1)
CC7	HDP7707, HDP7680, HDP8859	3 (3.4)	LAM1 (1), LAM2 (1), LAM9 (1)
CC8	HDP8796, HDP7711	2 (2.3)	T1 (1), Haarlem3/T1 (1)
CC9	HDP8045, HDP8015	2 (2.3)	T1 (2)
CC10	HDP8719, HDP7874	2 (2.3)	T3/X3 (1), LAM6 (1)
CC11	HDP8814, HDP8764	2 (2.3)	LAM3 (2)
CC12	HDP7803, HDP7937	2 (2.3)	LAM9 (2)

**Table 5:** Distribution of genotypic groups, MIRU-VNTR Clonal Complexes and SIT, through the Kilamba-Kiaxi and Viana municipalities.

Genotypic Group	No. of Isolates (%)	
	Kilamba-Kiaxi	Viana
Clonal Complex^1^		
CC1 (LAM1)	9 (26.47)	3 (15.79)
CC2 (LAM9)	2 (5.88)	2 (10.53)
CC3 (T1)	5 (14.71)	1 (5.26)
CC4 (T1)	1 (2.94)	2 (10.53)
CC5 (T1)	1 (2.94)	1 (5.26)
CC6	1 (2.94)	0 (0)
CC7	1 (2.94)	0 (0)
CC8	1 (2.94)	0 (0)
C9 (T1)	0 (0)	2 (10.53)
CC10	1 (2.94)	0 (0)
CC11 (LAM3)	12 (35.29)	8 (42.11)
Total	34 (100)	19 (100)
Clade		
HAARLEM3/T1	1 (2.94)	0 (0)
LAM1	9 (26.47)	2 (10.53)
LAM1/LAM9	1 (2.94)	0 (0)
LAM10	1 (2.94)	0 (0)
LAM3	2 (5.88)	0 (0)
LAM4	2 (5.88)	1 (5.26)
LAM6	2 (5.88)	1 (5.26)
LAM8	2 (5.88)	0 (0)
LAM9	5 (14.71)	4 (21.05)
T1	9 (26.47)	9 (47.37)
T2	0 (0)	1 (5.26)
T3/X3	0 (0)	1 (5.26)
Total	34 (100)	19 (100)
SIT		
144	1 (2.94)	2 (10.53)
1530	0 (0)	0 (0)
1548	1 (2.94)	0 (0)
1894	0 (0)	0 (0)
20	9 (26.47)	2 (10.53)
2025	1 (2.94)	0 (0)
244	2 (5.88)	2 (10.53)
290	1 (2.94)	0 (0)
306	0 (0)	1 (5.26)
33	1 (2.94)	0 (0)
42	4 (11.76)	4 (21.05)
52	0 (0)	1 (5.26)
53	4 (11.76)	3 (15.79)
60	2 (5.88)	1 (5.26)
635	1 (2.94)	0 (0)
64	1 (2.94)	1 (5.26)
95	1 (2.94)	0 (0)
Orphan	5 (14.71)	2 (10.53)
Total	34 (100)	19 (100)

^1^Spoligotyping clade in brackets was added to clonal complexes whenever these were composed by a clear majority of a specific clade.
