# Supplementary material for: Genetic diversity, transmission dynamics and drug resistance of Mycobacterium tuberculosis in Angola
Source: Sci Rep. 2017 Feb 23;7:42814. doi: 10.1038/srep42814 (PMC5322374; doi:10.1038/srep42814)
Supplement: Supplementary Tables [file srep42814-s1.pdf]

Supplementary Information

Genetic diversity, transmission dynamics and drug resistance of M*ycobacterium tuberculosis* in Angola

João Perdigão[1], Sofia Clemente[2], Jorge Ramos[3], Pedro Masakidi[2], Diana Machado[3], Carla Silva[1], Isabel Couto[3], Miguel Viveiros[3], Nuno Taveira[1,4*] and Isabel Portugal[1*]

[1]iMed.ULisboa – Instituto de Investigação do Medicamento, Faculdade de Farmácia, Universidade de Lisboa, Lisboa, Portugal;

[2] Hospital da Divina Providência, Serviço de Doenças Infecciosas, Luanda, Angola;

[3] Unidade de Microbiologia Médica, Global Health and Tropical Medicine, GHTM, Instituto de Higiene e Medicina Tropical, IHMT, Universidade Nova de Lisboa, UNL, Lisboa, Portugal;

[4]Centro de Investigação Interdisciplinar Egas Moniz, Instituto Superior de Ciências da Saúde Egas Moniz, Monte de Caparica, Portugal.

* NT and IP are joint senior authors.

Corresponding authors:

Isabel Portugal / Nuno Taveira

Instituto de Investigação do Medicamento, Faculdade de Farmácia, Universidade de Lisboa, Av. Prof. Gama Pinto, 1649-003 Lisboa, Portugal

Phone: +351 217946439, Fax: +351 217934212, E-mail: isabel.portugal@ff.ulisboa.pt; ntaveira@ff.ulisboa.pt

Supplementary Table 1 – Demographic and clinical indicators for the sample of patients enrolled in the present study.

Indicator	No. of Isolates (%)
**Gender**	
Male	53 (61.6)
Female	33 (38.4)
**Total**	**86 (100.0)**
Unknown	2 (2.2)
**Inhabitancy (Municipality)**	
Kilamba-Kiaxi	34 (58.6)
Rangel	1 (1.7)
Cacuaco	1 (1.7)
Viana	19 (32.8)
Cazenga	2 (3.4)
Samba	1 (1.7)
**Total**	**58 (100.0)**
Unknown	31 (34.8)
**TB Case Type**	
New	53 (88.3)
Relapse	3 (5.0)
Treatment Failure	2 (3.3)
Previously Treated	2 (3.3)
**Total**	**60 (100.0)**
Unknown	29 (32.6)
**Cavitary Disease**	
Yes	29 (58.0)
No	21 (42.0)
**Total**	**50 (100.0)**
Unknown	39 (43.8)
**HIV Co-infection**[1]	
Positive	8 (14.5)
Negative	47 (85.5)
**Total**	**55 (100.0)**
Unknown	34 (38.2)
**Treatment Regimen**[2]	
2HRZE/4HR	51 (87.9)
2HRZES/4HR	7 (12.1)
**Total**	**58 (100.0)**
Unknown	31 (34.8)
**Outcome**	
Cured	39 (65.0)
Treatment Completed	4 (6.7)
Default	4 (6.7)
Treatment Failure	2 (3.3)
Death	1 (1.7)
Transferred	10 (16.7)
**Total**	**60 (100.0)**
Unknown	29 (32.6)

[1] HIV-Co-infection at time of diagnosis;
[2] Drugs: H – INH, R – RIF, Z – PZA, E – EMB, S – STP. Number preceding the drug regimen indicates the duration of the intensive and continuation phase, respectively.

Supplementary Table 2 – Drug resistance profile and, spoligotyping and MIRU-VNTR-based classifications obtained for all clinical isolates studied.

Isolate ID	Drug Resistance Profile[1]	Spoligotyping		MIRU-VNTR	
		SIT	Clade	MIT	Clonal Complex
HDP7503	Susceptible	244	T1	Orphan	CC5
HDP7508	Susceptible	53	T1	389	Singleton
HDP7547	Susceptible	Orphan	LAM9	743	Singleton
HDP7582	R	1894	LAM9	Orphan	Singleton
HDP7603	IRS	20	LAM1	10	CC1
HDP7609	IRSEP	20	LAM1	10	CC1
HDP7617	Susceptible	1530	LAM4	Orphan	Singleton
HDP7626	Susceptible	20	LAM1	10	CC1
HDP7630	Susceptible	42	LAM9	Orphan	CC2
HDP7665	Susceptible	42	LAM9	Orphan	Singleton
HDP7666	I	244	T1	Orphan	Singleton
HDP7680	Susceptible	42	LAM9	Orphan	CC7
HDP7687	Susceptible	Orphan	LAM10	10	CC3
HDP7689	Susceptible	20	LAM1	10	CC1
HDP7692	Susceptible	42	LAM9	Orphan	Singleton
HDP7700	Susceptible	244	T1	Orphan	Singleton
HDP7702	Susceptible	144	T1	236	CC4
HDP7707	Susceptible	20	LAM1	Orphan	CC7
HDP7710	Susceptible	60	LAM4	Orphan	Singleton
HDP7711	Susceptible	2025	HAARLEM3:T1	770	CC8
HDP7718	Susceptible	53	T1	402	CC3
HDP7719	Susceptible	53	T1	Orphan	Singleton
HDP7730	Susceptible	Orphan	T1	Orphan	Singleton
HDP7731	IEP	2271	LAM2	10	CC1
HDP7732	S	42	LAM9	10	CC1
HDP7735	Susceptible	20	LAM1	298	CC1
HDP7737	I	42	LAM9	601	CC6
HDP7753	Susceptible	64	LAM6	601	CC6
HDP7762	Susceptible	1548	LAM8	Orphan	Singleton
HDP7766	S	53	T1	402	CC3
HDP7768	Susceptible	1535	LAM9	Orphan	CC2
HDP7803	Susceptible	42	LAM9	Orphan	CC12
HDP7808	Susceptible	42	LAM9	Orphan	Singleton
HDP7812	Susceptible	1321	LAM1:LAM4	Orphan	Singleton
HDP7816	I	20	LAM1	10	CC1
HDP7819	Susceptible	42	LAM9	803	Singleton
HDP7844	S	42	LAM9	Orphan	CC2
HDP7869	Susceptible	33	LAM3	38	Singleton
HDP7870	Susceptible	42	LAM9	Orphan	CC2
HDP7874	Susceptible	64	LAM6	Orphan	CC10
HDP7893	Susceptible	53	T1	402	CC3
HDP7899	Susceptible	42	LAM9	Orphan	Singleton

HDP7900	Susceptible	20	LAM1	10	CC1
HDP7909	Susceptible	144	T1	339	CC4
HDP7937	Susceptible	42	LAM9	Orphan	CC12
HDP7940	Susceptible	95	LAM6	Orphan	Singleton
HDP7942	Susceptible	42	LAM9	Orphan	CC2
HDP7946	Susceptible	Orphan	LAM9	Orphan	Singleton
HDP7955	IEP	20	LAM1	Orphan	CC1
HDP7980	Susceptible	244	T1	Orphan	CC5
HDP7984	Susceptible	Orphan	LAM1:LAM9	10	CC1
HDP7986	IS	74	T1	Orphan	Singleton
HDP8014	Susceptible	20	LAM1	10	CC1
HDP8015	Susceptible	53	T1	8	CC9
HDP8033	Susceptible	42	LAM9	601	CC6
HDP8039	Susceptible	144	T1	339	CC4
HDP8044	Susceptible	144	T1	339	CC4
HDP8045	Susceptible	53	T1	8	CC9
HDP8053	Susceptible	53	T1	402	CC3
HDP8054	Susceptible	1548	LAM8	Orphan	Singleton
HDP8055	Susceptible	60	LAM4	Orphan	Singleton
HDP8058	Susceptible	64	LAM6	Orphan	Singleton
HDP8627	I	244	T1	Orphan	CC5
HDP8628	Susceptible	20	LAM1	298	CC1
HDP8631	Susceptible	Orphan	T1	Orphan	Singleton
HDP8633	Susceptible	20	LAM1	10	CC1
HDP8635	IRS	52	T2	Orphan	Singleton
HDP8643	Susceptible	53	T1	402	CC3
HDP8644	S	1755	LAM6	Orphan	CC1
HDP8671	Susceptible	60	LAM4	Orphan	Singleton
HDP8719	Susceptible	Orphan	T3:X3	Orphan	CC10
HDP8729	IS	Orphan	LAM1:LAM9	Orphan	Singleton
HDP8754	Susceptible	2073	LAM3	Orphan	Singleton
HDP8756	Susceptible	Orphan	T1	Orphan	Singleton
HDP8764	Susceptible	635	LAM3	Orphan	CC11
HDP8784	IS	144	T1	Orphan	CC4
HDP8788	Susceptible	244	T1	Orphan	CC5
HDP8796	Susceptible	53	T1	770	CC8
HDP8813	I	20	LAM1	Orphan	Singleton
HDP8814	Susceptible	635	LAM3	Orphan	CC11
HDP8819	IS	20	LAM1	128	CC1
HDP8853	Susceptible	53	T1	558	Singleton
HDP8859	IREP	194	LAM2	130	CC7
HDP8886	Susceptible	53	T1	257	Singleton
HDP8907	I	20	LAM1	Orphan	Singleton
HDP8912	Susceptible	306	T1	Orphan	Singleton
HDP9020	S	20	LAM1	10	CC1
HDP9025	Susceptible	290	LAM8	Orphan	CC2

[1] Drug Resistance Profile: I, isoniazid; R, rifampicin; S, streptomycin; E, ethambutol; P, pyrazinamide.
